# Validity and reliability of HOP-Up: a questionnaire to evaluate physical activity environments in homes with preschool-aged children

**DOI:** 10.1186/s12966-016-0417-3

**Published:** 2016-08-18

**Authors:** Carolyn Cheng, Jennifer Martin-Biggers, Virginia Quick, Kim Spaccarotella, Carol Byrd-Bredbenner

**Affiliations:** 1 Rutgers, The State University of New Jersey, 26 Nichol Avenue, New Brunswick, NJ 08901 USA; 2 Kean University, 1000 Morris Ave, Union, NJ 07083 USA

**Keywords:** Physical activity, Environment, Neighborhood, Yard, Home, Children, Parent, Availability, Accessibility, Space, Questionnaire

## Abstract

**Background:**

Early identification of physical activity (PA) opportunities in the home and neighborhood environment may help obesity prevention efforts in households with young children. This cross-sectional study’s purpose was to develop a brief, easy-to-use, self-report inventory called Home Opportunities for Physical activity check-Up (HOP-Up), to evaluate the availability and accessibility of PA space and equipment in and near homes with preschool children, and establish its validity and reliability.

**Methods:**

The HOP-Up was field tested by two trained researchers and parents of preschool-aged children (*n* = 50; 71 % white). To establish criterion validity, researchers were the ‘gold standard’ and visited participants’ homes to assess their PA environments using the HOP-Up, while participants separately completed their HOP-Up. Two weeks later, parents completed the HOP-Up online for test-retest reliability. After minor survey refinements, exploratory and confirmatory factor analysis using a split-half cross validation procedure was conducted in a larger sample of participants (*n* = 655, 60 % white) who completed the HOP-Up online to examine its factor structure. To establish convergent validity, correlations were conducted to compare HOP-Up scales from the factor solution generated with PA behavior and cognitions, and reported screen time.

**Results:**

Intra-class correlations (ICCs) examining HOP-Up item agreement between researcher and parents revealed slight to substantial agreement (range 0.22 to 0.81) for all items. ICCs for all HOP-Up items ranged from fair to substantial agreement between parent responses at both time points (range 0.42 to 0.95). Exploratory factor analysis revealed a five factor solution (18 items), supported eigen values, scree plots, review for contextual sense, and confirmatory factor analysis. Additionally, there were significant (*p* < 0.05) positive correlations among nearly all five HOP-Up scales with parent and child physical activity levels (range 0.08 to 0.35), and values parents placed on PA for self and child (range 0.16 to 0.35), and negative correlations of Neighborhood Space & Supports for PA scale with parent and child reported screen time (*r* = −0.11, *r* = −0.13, respectively).

**Conclusions:**

Findings support the psychometric properties of this brief, easy-to-use, HOP-Up questionnaire, which may help parents, prevention researchers, residential planners, and practitioners increase their understanding of how the home environment—inside, outside, and the neighborhood— impacts preschool children’s physical activity levels.

## Background

Daily physical activity is key to maintaining a healthy lifestyle and preventing excess weight gain [1, 2]. According to the Social Cognitive Theory [3], behaviors, such as physical activity, and the environment interact simultaneously and reciprocally. Behavior can affect the environment, but the environment also shapes behavior [3]; consequently, changing the physical activity environment potentially could increase physical activity and reduce sedentary activity.

A barrier to being physically active is environments that are not supportive of physical activity. For instance, half of the children in the United States do not have a park, community center, and/or sidewalk in their neighborhood [4]. Perceptions of crime, traffic safety, and walkability among families with children and adolescents also influence use of physical activity supports (e.g., parks, playgrounds) [5]. Children residing in urban areas engage in significantly less physical activity than peers living in suburban areas, and urban parents express more anxiety about neighborhood safety [5–8].

Home environments supportive of physical activity are especially important for young children because of the proportion of time they spend at home. Children living in homes that provide many opportunities for sedentary behaviors, such as those with many televisions, computers, and other electronic media devices, may be less active [9] and get more than the recommended 2 h of screen time daily [10–13]. Additionally, screen time tends to increase energy intake by promoting snacking behavior and suppressing satiety cues during screen time [14, 15]. Evidence also indicates that having physical activity equipment in the home is negatively correlated with TV viewing [16], and parents’ physical activity, support, and time spent with child are positively correlated with their children’s physical activity levels [17, 18].

Equally important to supporting physical activity are access to equipment, programs, and space [19]. Accessibility may help to promote “ease of use and cueing of behavior,” [20] and is thus, important as a prompt to engage in specific behaviors (e.g., use of available equipment). In a prior review, access to facilities and physical activity programming was related to increased children’s physical activity [19] and may be an important predictive barrier to physical activity among adults [16], who model physical activity behaviors for their children.

Early identification of the availability and accessibility of physical activity opportunities in the home and neighborhood environment may help childhood obesity prevention efforts. Several studies have focused on the neighborhood physical activity environment [2, 21–23]; however, little is known about physical activity opportunities in and around the outside of homes where families with young children reside. In addition, many previous home environment assessments have not considered the frequency with which available physical activity supports are accessed [24]. Furthermore, existing questionnaires for assessing home physical activity environments tend to be burdensome (lengthy, difficult to use), not well matched to households with preschool children, and/or do not report psychometric data [16, 20, 25–32]. Few existing questionnaires are validated and/or have established reliability [20, 25, 27, 28, 32]. The variability and lack of standard questionnaires specific to households with preschoolers make published data difficult to compare [2, 16, 20–23, 25–33]. Thus, the purpose of this study was to develop a brief, easy-to-use, self-report inventory to evaluate the availability and accessibility of physical activity space and equipment in and near homes with preschool children between the ages of 2 and 5 years and to establish the validity and reliability of the questionnaire.

## Methods

The HOP-Up (Home Opportunities for Physical activity check-Up) questionnaire aimed to assess the availability of physical activity equipment and space inside the homes, immediately outside the homes (yard), and in the neighborhoods of families with preschool-aged children and how frequently equipment and space are accessed. Ethical approval to conduct this study was granted by the institutional review board at Rutgers University.

### Questionnaire development

Questionnaire development was a ten-phase process (Table 1). The first phase was an extensive examination of published questionnaires designed to assess physical activity supports and frequency of access in the home, immediately outside the home (yard), and in the neighborhood to identify key components to incorporate in the study questionnaire [2, 16, 20–23, 25, 26, 28, 29, 31, 34]. The second phase involved creating a bank of items from the review conducted in the first phase that, based on this study’s purpose, could be used or adapted and organized by location in and around the home (i.e., inside the home, immediately outside the home, and neighborhood). Items were derived from numerous published questionnaires [23, 25–27, 31, 35–38].

**Table 1:** The 10-phases of the HOP-Up physical activity environment questionnaire development

Phase 1 Literature Review
▪ Extensive review of published questionnaires that assess physical activity and/or sedentary activity supports and frequency of use in the home to identify key components of the home and near environments related to obesity risk
Phase 2 Item Bank Creation
▪ Identification of items pertinent to study purpose
▪ Adaptation, enhancement, and expansion of questionnaire items
▪ Organization of items by location category (i.e., inside the home, immediately outside the home [i.e., yards], and neighborhoods [i.e., playgrounds])
Phase 3 Initial Expert Review of Items
▪ Expert review (*n* = 6) of items to identify appropriateness, gaps in assessment, and suitability for use in homes with preschool children
Phase 4 Item Refinement
▪ Further item adaptation, expansion, and *de novo* development
▪ Revision or elimination of items that were age-inappropriate (e.g., presence of basketball hoop at the home)
Phase 5 Questionnaire Design
▪ Creation of questionnaire layout
▪ Development of scoring procedures
Phase 6 Content Validity Review
▪ Second expert (*n* = 8) review and refinement to establish content validity and confirm scoring procedure
▪ Refinement of items
Phase 7 Cognitive Testing
▪ Cognitive testing conducted with parents of preschoolers (*n* = 5) to ensure accurate comprehension and establish face validity
▪ Refinement of items
Phase 8 Field Testing to Establish Criterion Validity and Test-Retest Reliability
▪ Field-tested questionnaire with parents age ≥18 and <45 years and having at least one child 2- to 5-years-old
▪ Part 1: At home visits, parents and researchers simultaneously, but independently, completed the questionnaire (researchers served as the “gold standard” or criterion and followed specific guidelines)
▪ Part 2: ~2 weeks later, parents completed the questionnaire online again to establish test-retest reliability
▪ Refinement of the questionnaire based on field testing results.
Phase 9 Establishing Scale Unidimensionality, Internal Consistency, and Convergent Validity
▪ Factor analysis of the refined questionnaire with parents (*N* = 655) having at least one child 2- to 5-years-old completing baseline questionnaires for the HomeStyles intervention.
▪ Split-half cross-validation with exploratory (*n* = 327) and confirmatory (*n* = 330) halves to examine factor structure of the measure using Principal Components Analysis with varimax rotation (orthongonal) and Kaiser normalization to establish unidimensionality of the scales.
▪ Part 1: Iterative exploratory factor analysis conducted to identify strong factor solutions (minimum loading of 0.4) and eliminate cross-loading items (loadings on >1 scale within <0.2 of each other).
▪ Part 2: Verify exploratory factor solutions via confirmatory factor analysis.
▪ Calculate internal consistency of final scales.
▪ Test convergent validity of the final scales in parents and children with other physical activity measures.
Phase 10 Final Expert Review
▪ Expert review (*n* = 5) of items to confirm content validity of the final questionnaire.

In phase three, a panel of experts in tests and measurements, physical activity, and community-based obesity prevention programs (*n* = 6) reviewed the bank of items to identify those assessing each key component, gaps and overlaps in items, and suitability of items for use in homes with preschool children. In the fourth phase, researchers developed, adapted, and/or expanded items to address gaps and eliminated or collapsed overlapping items. Researchers also revised or eliminated items that did not pertain to physical activities that were age-appropriate for preschool children (e.g., presence of basketball hoop at the home). Guidelines for development of instrument items were carefully followed throughout the development and refinement of the questionnaire [39–41].

In phase five, items were formatted and scoring procedures established. In phase six, the questionnaire underwent review by a panel of experts (*n* = 8) in tests and measurements, physical activity, and community-based obesity prevention programs to establish content validity and further refine the items. Experts were instructed to determine whether the items comprehensively reflected the key environmental factors in and around the home that could affect activity levels of preschool-aged children. Experts felt that the questionnaire adequately reflected the key environmental factors, such as space and active play supports related to physical activity levels of preschool-aged children in and around the home and agreed the questionnaire had content validity. They had minor suggestions for streamlining the questionnaire.

In phase seven, the questionnaire was subjected to cognitive testing [42] to ensure that the parents of preschoolers (*n* = 5) interpreted the items as intended and to establish face validity. During cognitive testing, parents were asked to read each item aloud, describe in their own words what the question was asking, and then explain how they would answer the question using the response choices provided. Researchers assessed results to determine how accurately participants understood the question and response choices. Cognitive testing results and suggestions from parents were used to refine the questionnaire. Refinements included improvements in instructions, adding definitions of active play to each section of the questionnaire (i.e., active play means doing activities that make the child sweat and breathe harder than normal, like riding scooters or tricycles, running, dancing, jumping, and horseplay or wrestling), and incorporation of bolding, italicizing, and underlining to emphasize keywords, such as *inside* [the home], *outside* [the home], *neighborhood*, and *active play* to reinforce the question to help ensure accurate responses.

In phases 8, 9, and 10, the questionnaire was field tested, tested with a large sample of preschool parents, and subjected to a final expert review, respectively. The focus of this article is to report on the last three phases of development (i.e., phases 8 to 10).

### Phase 8

The goal of the phase 8 was to field test the HOP-Up questionnaire and establish criterion validity and test-retest reliability. HOP-Up evaluated the physical activity environment in three main locations: inside the home, area right outside the home (yard), and neighborhood. Table 2 shows the items and organization of the questionnaire used in phase 8. The phase 8 version of the *Physical Activity Environment Inside the Home* section of the questionnaire had three scales. The *Availability of Space & Supports Inside* scale assessed space available inside the home for active play (2 items) and supports for physical activity inside (e.g., toys, active video games, playmates) (3 items). The *Accessibility Inside* scales evaluated parent policy related to time permitted for active indoor play (1 item) and the ease with which preschool children can access indoor play supports and play indoors without help from parents (2 items). *Availability of Space & Supports Inside* and *Accessibility Inside* scales had a 5-point Likert scale (i.e., strongly disagree to strong agree). The 3-item *Frequency of Inside Active Play* scale measured how often preschool children play inside, use physical activity supports, and play with siblings or friends. Answer choices were a 5-point scale ranging from almost never to every day.

**Table 2:** Phase 8-field testing results of the HOP-Up physical activity environment questionnaire: researcher and parent scores and intraclass correlations (ICCs)

SECTION Scale, *Subscale*, Item	Researcher Score	Parent Score	Parent Re-test	ICCs	ICCs	Cronbach’s α
	Mean ± SD	Mean ± SD	Mean ± SD	Parent vs Researcher	Parent vs Re-test	Parent Re-test
	*N* = 50	*N* = 50	*N* = 48			
Physical Activity Environment Inside the Home						
* Availability of Space & Supports Inside the Home*						0.66
My child has plenty of room for active play inside our home.^a^	4.06 ± 1.15	3.96 ± 1.03	3.94 ± 0.78	0.34	0.79	
My child has enough space inside our home to do somersaults and cartwheels without hitting furniture or walls.^a^	3.74 ± 1.17	3.54 ± 1.28	3.85 ± 1.13	0.47	0.79	
My child has plenty of toys for active play that can be used indoors to help build muscles. These are toys like balls, tricycles, and scooters.^a^	3.66 ± 1.24	3.98 ± 1.12	3.75 ± 1.02†	0.31	0.85	
My child has video games that help the child be active. These are video games played standing up and require lots of moving like Wii Fit, Xbox Kinect.^a^	2.40 ± 1.63	3.00 ± 1.51	2.85 ± 1.44	0.59	0.90	
My child has siblings or friends that live nearby to play with indoors. ^a^	§	4.42 ± 0.96	4.19 ± 1.00	--	0.68	
*Accessibility Inside the Home*						
I put limits on the amount of time my child can have active play indoors.^$a^	§	3.44 ± 1.35	3.42 ± 1.11	---	0.78	0.46
It’s easy for my child to actively play indoors without my help.^a^	§	4.23 ± 0.95	4.17 ± 0.78	--	0.54	
Indoor equipment for active play is stored where it is easy for my child to see and reach.^a^	4.84 ± 0.42	4.38 ± 0.75*	4.19 ± 0.76	0.29	0.45	
*Frequency of Inside Active Play Inside the Home*						0.71
How often does your child usually play actively inside your home?^b^	§	4.35 ± 1.00	4.21 ± 1.03	--	0.76	
How often does your child play actively indoors with toys that help build muscles?^b^ These are toys like balls, tricycles, scooters.	§	3.48 ± 1.37	3.25 ± 1.38	--	0.70	
How often does your child play actively indoors with siblings or kids that live nearby?^b^	§	3.71 ± 1.43	3.71 ± 1.44	--	0.81	
Physical Activity Environment Outside the Home (Yard) ^c^						
*Availability of Space & Supports Outside (Yard)*						0.82
The yard or area outside our home has plenty of room for my child to actively play games like tag or chase.^a^	4.90 ± 0.37	4.73 ± 0.59	4.68 ± 0.52	0.81	0.64	
There is a paved or flat area in the yard or area outside our home that is big enough for my child to safely ride a tricycle, bike, scooter, or other wheeled toy.^a^	4.65 ± 1.00	4.66 ± 0.61	4.61 ± 0.69	0.74	0.45	
Think about the size of parking spaces at the shopping mall. Now, think about all the areas outside your home where you would allow your child to play actively—include grassy, paved, or other areas. If those areas became a parking lot, about how many parking spaces would there be?^d^	4.65 ± 0.91	4.50 ± 1.03	4.53 ± 0.97	0.22	0.92	
The yard or area outside our home has plenty of swings, slides, or other active play equipment my child can use.^a^	2.98 ± 1.68	3.73 ± 1.45*	3.95 ± 1.31†	0.85	0.93	
My child has plenty of toys for playing actively outside, like balls, jump ropes, skates, swimming or kiddie pool, hula hoops, or sleds.^a^	3.52 ± 1.35	4.43 ± 1.02*	4.45 ± 0.85	0.62	0.80	
My child has a tricycle, bike, scooter, or other wheeled toy to use outside.^a^	4.75 ± 0.86	4.64 ± 0.81	4.55 ± 0.82	0.86	0.95	
My child has shoes and clothes for playing actively outside.^a^	4.96 ± 0.20	4.66 ± 0.89*	4.68 ± 0.71	0.31	0.84	
* Accessibility Outside (Yard)*						0.51
I often limit my child’s active play in the yard or area right outside our home.^*$a^	§	3.45 ± 1.32	3.14 ± 1.36	---	0.60	
It’s easy for my child to see and reach toys for playing actively outside.^a^	4.23 ± 1.15	4.13 ± 1.10	4.36 ± 0.87	0.30	0.81	
It’s easy for my child to actively play in the yard or area right outside our home without my help.^a^	§	3.91 ± 1.33	4.02 ± 1.11	--	0.80	
*Frequency of Outside (Yard) Home Play*						¶
How often does your child go on walks with the dog or play with it outside (doing things like throwing balls)?^e^	§	1.56 ± 1.17	1.52 ± 1.09	---	0.95	
When the weather is good, how often does your child usually play actively in the yard or area outside your home?^b^	§	4.11 ± 1.13	3.98 ± 1.13	---	0.88	
Physical Activity Environment in the Neighborhood						
* Availability of Space & Supports in the Neighborhood*						0.66
There are outdoor areas, like parks, pools, and playgrounds, nearby my home where kids can play actively.^f^	4.16 ± 0.68	4.46 ± 0.93	4.29 ± 0.97†	0.52	0.92	
There are free or low-cost recreation centers or other indoor places where kids can play actively.^f^	3.94 ± 1.51	3.49 ± 1.40*	3.25 ± 1.35	0.81	0.81	
The outdoor areas in my neighborhood have plenty of swing sets, slides, or other play equipment my child can use.^f^	4.26 ± 1.16	4.19 ± 1.23	4.15 ± 1.05	0.69	0.87	
*Neighborhood Access & Safety*						0.53
There is so much traffic near where I live that I do not feel safe walking in the area.^$a^	4.16 ± 1.08	4.13 ± 0.94	4.15 ± 0.74	0.62	0.75	
I feel safe from crime in my neighborhood and nearby.^a^	4.28 ± 1.18	4.00 ± 1.05	4.13 ± 0.89	0.57	0.53	
I feel safe from biting insects, like mosquitos, ticks, and scorpions, and animals, like dogs running loose, in my neighborhood and nearby.^a^	§	3.13 ± 1.24^g^	3.13 ± 1.17	---	0.78	
The outdoor areas in my neighborhood where my child can play actively are safe.^a^	4.34 ± 0.96	4.35 ± 0.73	4.21 ± 0.82	0.48	0.81	
The outdoor areas in my neighborhood where my child can play actively are clean.^a^	4.50 ± 0.81	4.23 ± 0.88	4.17 ± 0.83	0.62	0.76	
The outdoor areas in my neighborhood where my child can play actively are crowded with other people.^f^	§	2.14 ± 0.90^h^	2.34 ± 1.01	---	0.55	
In my neighborhood, it’s easy to get to outdoor areas where kids can play actively.^f^	4.50 ± 0.74	4.33 ± 0.78	4.27 ± 0.61	0.54	0.42	
*Frequency of Neighborhood Active Play*						0.73
When the weather is good, how often does your child usually play actively in outdoor areas, like parks, pools, and playgrounds, near your home?^b^	§	3.44 ± 1.30	3.19 ± 1.25	---	0.79	
How often does your child usually play actively in free or low-cost recreation centers or other indoor places near your home?^b^	§	1.77 ± 0.83	1.73 ± 0.87	---	0.76	

*Researcher score significantly different from parent score (*P* < .05) using independent t-tests

†Parent score significantly different from parent re-test (*P* < .05) using paired t-tests

§ Item could not be observed by researchers and thus could not be included in the criterion validity assessment

^$^ Reverse score item

¶ Too few families (*n* = 13) report having a dog, making intraclass correlations inappropriate

^a^Answer choices: Strongly disagree, disagree, neither agree nor disagree, agree, strongly agree; score = 1 to 5, respectively

^b^Answer choices: Almost never, 1–2 times/week, 3–4 times/week, 5–6 times/week, every day; score = 1 to 5, respectively

^c^The sample size for the Physical Activity Environment Outside the Home (Yard) items was 48 for researcher, 48 for parent, and 44 for parent retest because 2 individuals did not have space outside their home and 4 did not take the survey online

^d^Answer choices: 0 to 2 = 1, 3 to 4 = 2, 5 to 6 = 3, 7 to 8 = 4, ≥9 = 5

^e^Answer choice: 1 = No dog or Almost never walk/play with dog, 2 = 1 or 2 times a week walk/play with dog, 3 = 3 to 4 times a week walk/play with dog, 4 = 5 to 6 times a week walk/play with dog, 5 = Every day walk/play with dog)

^f^Answer choices: Strongly disagree, disagree, neither agree nor disagree, agree, strongly agree, don’t know; score = 1 to 5, respectively and items with “don’t know” answers were omitted from score

^g^ *n* = 47 because 1 parent did not respond to this item at the home visit 1 and another 2 parents did not complete the survey online (retest)

^h^ *n* = 44 because this item was added after completing 4 home visits and 2 parents did not complete the online survey (retest)

The *Physical Activity Environment Outside the Home* (*Yard*) section focuses on areas in the yard or areas immediately outside the home and has three scales analogous to those from the inside the home section of the questionnaire. The *Availability of Space & Supports Outside* (*Yard*) scale assessed space for active play (3 items) and supports for active play (e.g., swings and slides; toys like balls and wheeled toys, and play shoes and clothes; 4 items). The 3-item *Accessibility Outside* (*Yard*) scale appraised parent policy with regard to time allowed for playing outdoors and how easy it is for preschool children to access outdoor play equipment and to play outside without adult help. All items on the *Availability of Space & Supports Outside* (*Yard*) and *Accessibility Outside* (*Yard*) scales had 5-point Likert response choices ranging from strongly disagree to strongly agree, except for the item asking parents the size of the play area outside their homes which asked participants to estimate the equivalent number of parking spaces available for outside play. The *Frequency of Outside* (*Yard*) *Home Play* scale evaluated how often children played outside and played with a dog (2 items). The *Frequency of Outside* (*Yard*) *Home Play* items had the same answer choices as the *Frequency of Inside Active Play* scale.

Similar to the other two sections of the questionnaire, the *Physical Activity Environment in the Neighborhood* section also had three analogous scales. The *Availability of Space & Supports in the Neighborhood* scale assessed space availability for active play (e.g., parks, pools, playgrounds, recreation centers; 2 items) and active play supports (e.g., swing sets, slides, other play equipment for children; 2 items). The *Neighborhood Access & Safety* scale assessed neighborhood safety (e.g., traffic, crime, biting insects, crowding; 6 items) and ease with which neighborhood play areas can be accessed (1 item). Answer choices for items on these scales were a 5-point Likert scale ranging from strongly disagree to strongly agree; however, a “don’t know” choice was available in case parents were not well acquainted with their neighborhood. The 2-item *Frequency of Neighborhood Active Play* scale assessed how frequently neighborhood active play areas were used and had the same answer choices as used on analogous scales for inside the home and outside (yard) areas.

All Physical Activity Environment items with a 5-point Likert scale were scored as 5, 4, 3, 2, or 1 points if answered strongly agree, agree, neither agree nor disagree, disagree, or strongly disagree, respectively, when the item had a positive polarity. Scoring was reversed for items with a negative polarity. “Don’t know” answer choices were not scored. Frequency items were scored as 5, 4, 3, 2, or 1 points for answers every day, 5 to 6 times a week, 3 to 4 times a week, 1 or 2 times a week, or almost never, respectively. The item estimating physical activity space in the yard (in terms of parking spaces) was scored as follows: 1 = 0 or 1 space, 2 = 2 to 3 spaces, 3 = 4 to 5 spaces, 4 = 6 to 7 spaces, 5 = more than 7 spaces. Scores for each item in a scale were averaged to create the scale score. Higher scores indicated greater availability of space, greater availability of active play supports, more positive parent policies toward active play, easier accessibility to physical activity, greater sense of safety for active play, and greater frequency of active play.

#### Phase 8 sample

In the phase 8 field-test (summer/fall 2013), parents of preschool-aged children living in New Jersey were recruited by multiple electronically mailed announcements, word of mouth, and notices distributed in daycare centers and preschools in central New Jersey. The recruitment advertisement included a link to the eligibility screener. Eligibility criteria included ≥18 and <45 years of age and at least one preschool child (i.e., 2- to 5-years old) along with consenting to participate.

#### Phase 8 design

The phase 8 field test had two parts. In part one, two trained researchers visited the homes of participants. Participants were asked to make no changes to their home environments prior to the visit. During the home visit, researchers and parents simultaneously, but independently, assessed the physical and sedentary activity environment of parents’ home environments using a pencil and paper format. Parents completed the entire phase 8 questionnaire and researchers completed only the items that could be readily observed (see Table 2). Researchers assessed the neighborhood using online resources (e.g., Google Maps) and conducted visual observations upon arrival into the neighborhood. To ensure uniformity and objectivity in answering items across households, researchers’ responses to the questionnaire were based on a set of specific criteria (e.g., for items related to “plenty of toys”, researchers counted total toys and selected among strongly disagree to strong agree answer choices based on the number of toys set equivalent to the answer choice).

The purpose of part one in phase 8 was to allow researchers to serve as the “gold standard” or comparison for parent responses in order to establish criterion validity of the questionnaire. In part two of the study, parents completed the questionnaire two weeks later using an online format. The purpose of part two (re-test) was to compare parent responses with part one (test) responses to determine test-retest reliability of the questionnaire. Parents were compensated $20 for completing part one and $30 for part two of the study.

#### Phase 8 data analysis

Data were analyzed with IBM SPSS Statistics 22.0 (SPSS, Chicago, IL). For part 1 of phase 8, mixed effects with consistency agreement intraclass correlation coefficients (ICC) between each item completed by parents and researchers at the home visit were calculated to assess criterion validity. Additionally, independent t-tests between researcher and parent scores were conducted to assess significant differences of survey items. To determine test-retest reliability (phase 8, part 2), parents completed the questionnaire independently during the home visit (test) and again 2 weeks later (re-test) as an online survey. Significance was set at *P* < 0.05. ICCs for the test and re-test responses were calculated for each item to determine agreement. Paired t-tests were conducted to determine whether significant differences occurred between test and re-test responses. Internal consistency for all scales of the parent re-test online survey was assessed using Cronbach’s alpha. For all ICCs, agreement of data was assigned ratings using guidelines proposed by Shrout’s cut-off points (i.e., ICC < 0.10 = virtually none, 0.11–0.40 = slight, 0.41–0.60 = fair, 0.61–0.80 = moderate, 0.81–1.0 = substantial) [43].

### Phase 9

This phase commenced after refining the HOP-Up questionnaire using phase 8 results. Phase 9 served to establish scale unidimensionality, internal consistency, and convergent validity of the refined HOP-Up questionnaire.

#### Phase 9 sample

In phase 9 (spring 2014 to summer 2015), parents of preschool children were recruited via electronically mailed announcements, and verbal recruitment efforts to participate in the HomeStyles healthy family living intervention. Eligible parents were between the ages of 20 and 45 years and had at least one preschool child.

#### Phase 9 design

Phase 9 data were collected via an online cross-sectional survey. Parents that consented and completed the entire baseline assessment survey online were compensated $15.

The survey included the HOP-Up questionnaire, as well as scales assessing physical activity behaviors and cognitions. Physical activity was assessed by a physical activity index for the parent and their preschool child that was built on the activity categories (i.e., vigorous, moderate, and walking) defined by the International Physical Activity Questionnaire [44]. This index is based on the days per week (0 to 7 days) vigorous activities (e.g., heavy lifting, digging, aerobics; activities that make an individual sweat or breathe a lot harder than usual), moderate activities (e.g., carrying light loads, bicycling at a regular pace; activities that make an individual sweat or breathe a little harder than usual), and walking for at least 10 min at a time are completed [45]. The index score is calculated as follows: (#days/week of vigorous activities × 3) + (# days/week of moderate activities × 2) + (# days/week of walking ≥10 min at a time). Vigorous activity is weighted higher than other types of activities given differences in calorie expenditure of these activities. Possible score range is 0 to 42 with higher scores indicating greater physical activity.

Physical activity behavior and cognitions were assessed with five Likert scales. The value parents placed on physical activity for themselves, value they placed on physical activity for their preschool children, importance placed on not modeling sedentary activity to their preschool children, and outcomes expected as a result of engaging in physical activity were assessed using scales with 3, 2, 2, and 6 items, respectively [36, 37]. Answer choices for these four scales ranged from 1 to 5 (strongly disagree to strongly agree). For all four scales, items were averaged for an overall score with higher scores indicating greater importance placed on physical activity for self, physical activity for child, and not modeling sedentary behaviors and more positive outcomes expected from engaging in physical activity. The fifth scale measured the frequency with which parents modeled moderate and vigorous physical activity for their child. Responses to this 2-item scale were on a 7-point scale ranging from almost never to everyday [25–27, 31]. Items were averaged for an overall score with higher scores indicating greater frequency of modeling physical activity for their preschool children. The survey also captured total parent screen time daily and amount of screen time parents permitted preschool children to have daily (i.e., TV/movie time, sedentary computer/media device time, and active video game time).

#### Phase 9 data analysis

A split-half cross validation procedure was conducted by randomly splitting the sample into exploratory and confirmatory factor analysis halves using the random selection procedure in SPSS 22.0 (SPSS, Chicago, IL). Exploratory factor analysis was performed via iterative Principal Components Analysis with varimax rotation (orthogonal) and Kaiser normalization, and commenced with all items included. Factor loadings were examined after each analysis with one item at a time being removed in an iterative fashion. Items were retained based on factor loading strength (i.e., ≥0.4), not cross loading (i.e., cross loading defined as loading on >1 factor within <0.2 of each other), and whether the strongest loading made contextual sense with regard to other items strongly loading on the same factor [46]. This process was repeated until each remaining item was contextually similar to other items strongly loading on the same factor, total number of factors were in agreement with the sharpest turn in the slope of the scree plot, factor eigen values exceeded 1, and each factor generated an acceptable Cronbach’s alpha coefficient.

In the confirmatory factor analysis, the final factors remaining in the exploratory factor analysis were subjected to the same factor analysis procedures used as in the exploratory factor analysis to confirm the unidimensionality of the scale items. Cronbach’s alpha coefficients of internal consistency were calculated for each factor in the confirmatory analysis and for the combined split-half samples. To establish convergent validity, Spearman rank-order correlations were conducted to compare HOP-Up scales with physical activity behavior and cognitions as well as screen time measures.

### Phase 10

A final expert review of the factor solutions generated in phase 9 was conducted in Fall 2015 to confirm scale content validity. Five experts in tests and measurements, physical activity, and community-based obesity prevention programs conducted this review.

## Results and discussion

### Phase 8 field test

A total of 128 parents responded to recruitment notices for the phase 8 field test, with 104 completing the eligibility screener. Of those, 97 were eligible; parents living within 30 miles of the study site (*n* = 67) were invited to participate and the remainder (*n* = 30) were placed on a waiting list. Home visits were scheduled as soon as eligible parents accepted the participation invitation until the study quota of 50 homes was reached. Home visits took a maximum of 45 min to complete. Participants were mainly non-Hispanic, white (71 %) or Hispanic (19 %), female (94 %), had completed at least some college (90 %), and were overweight or obese (48 %).

A comparison of parent and researcher responses to each item on the *Physical Activity Environment Inside the Home* scale revealed ICCs ranging from 0.29 to 0.59, indicating slight to fair agreement (see Table 2). Researcher and parent scores did not differ significantly for any item, except the item assessing how easy it is for child to see and reach stored indoor equipment for active play.

A comparison of the *Physical Activity Environment in the Area Immediately Outside the Home* (*Yard*) items showed slight to substantial agreement between parents and researchers (range = 0.22 to 0.86). Researcher mean scores for two items related to equipment and toys to support active play outside were significantly lower than parents whereas researcher scores for the item related to outdoor clothing and shoes was significantly higher than parent scores.

Researcher and parent score ICCs for the *Physical Activity Environment in the Neighborhood* items showed fair to substantial agreement (range = 0.48 to 0.81). There were no significant differences between researcher and parent scores for any of the items, except for one item where researchers scored significantly (*p* < 0.05) higher on rating whether there are free or low-cost recreation centers or other indoor places for kids to play compared to parents.

In part 2 of phase 8, ICCs for all physical activity items ranged from fair to substantial agreement between parent responses at both time points (test-retest). Paired t-tests indicated few significant differences between test and retest scores (see Table 2).

Part 1 and 2 results from phase 8 were scrutinized to identify needed refinements. An examination of items with ICCs below the fair threshold and/or differing significantly between parent and researchers indicated they were similar to prior research [25]. That is, the specificity and objectivity of instructions for assigning scores that were given to researchers may have been incongruent with parent perceptions. For instance, parent scores for two items on the *Availability of Space & Supports Outside* (*Yard*) (“The yard or area outside our home has plenty of swings…” and “My child has plenty of toys for playing…”) were significantly higher than researchers, suggesting that perceptions of what constitutes “plenty” to parents differed from instructions given to researchers. Phrasing of these items was reviewed and six items were revised to increase clarity. Five items associated with the ease with which children can actively play without adult help and access play supports were deleted because they were highly correlated with frequency of play items, which are proxies for ease of access and independent play. Another item on availability of low-cost recreation centers for kids to play actively also was deleted as it was significantly highly correlated with the frequency of going to these low-cost recreation facilities for indoor play.

### Phase 9 establishing scale unidimensionality, internal consistency, convergent validity, and readability

A total of 655 parents completed the refined HOP-Up questionnaire and were randomly split into two groups: 327 for exploratory factor analysis and 330 for confirmatory factor analysis. Participants were mostly non-Hispanic, white (60 %) or Hispanic (21 %), in their 30’s (age 32.63 ± 5.70SD years), female (92 %), and nearly half had a college degree. There were no significant sociodemographic differences between the exploratory and confirmatory factor analysis participants.

The item related to frequency of playing with the family dog was eliminated from analysis prior to beginning exploratory factor analysis because a substantial number of participants (55 %) did not have a dog. Exploratory factor analysis indicated that 9 items did not meet the criteria for retention described in Data Analysis. As shown in Table 3, the final exploratory factor analysis results revealed a five factor solution, supported eigen values, scree plots, a review for contextual sense, and the confirmatory factor analysis. The 18 items in the five factor solution had loadings ranging from 0.43 to 0.88 for the exploratory factor analysis and 0.44 to 0.88 for the confirmatory factor analysis.

**Table 3:** Factor loadings from the exploratory and confirmatory factory analysis of the HOP-Up physical activity environment questionnaire

Scale	Exploratory Factory Analysis Scales					Confirmatory Factory Analysis Scales				
Item	(*n* = 327)					(*n* = 330)				
	A	B	C	D	E	A	B	C	D	E
**Scale A: Indoor Home Space & Supports for Physical Activity**										
My preschool kids have plenty of room to run around and burn off energy inside our home.^a^	0.62					0.62				
Think about the areas inside the home where your kids run around and burn off energy. How many somersaults or cartwheels can they do in a row without hitting furniture?^c^	0.76					0.61				
Think about all balls, tricycles, bicycles, scooters, jump ropes, and toys that help your preschool child run around and burn off energy inside your home. How many of these does your child have?^b^	0.43					0.44				
How often do your preschool kids run around and burn off energy inside your home?^d^	0.79					0.77				
How often do your preschool kids play indoors with balls, tricycles, bicycles, scooters, and other play things that help to burn off energy?^d^	0.78					0.69				
How often do your preschool kids run around and burn off energy indoors with siblings or kids who live nearby?^d^	0.57					0.54				
**Scale B: Outdoor/Yard Space & Supports for Physical Activity**										
The yard or area outside our home has plenty of room for my preschool kids to actively play games like tag or chase.^a^		0.75					0.82			
There is a paved or flat area in yard or area outside our home that is big enough for my preschool kids to safely ride a tricycle, bike, scooter, or other wheeled toy.^a^		0.76					0.70			
My preschool kids have shoes and clothes for playing actively outside.^a^		0.66					0.74			
My preschools kids have plenty of toys for playing actively outside like balls, jump ropes, skates, swimming or kiddie pool, hula hoops, or sleds.^a^		0.69					0.81			
**Scale C: Neighborhood Space & Supports for Physical Activity**										
There are outdoor areas like parks, pools and playgrounds nearby my home where my preschool kids can play actively.^e^			0.84					0.88		
The outdoor areas in my neighborhood have plenty of swings sets, slides or other play equipment my preschool kids can use.^e^			0.85					0.84		
The outdoor areas in my neighborhood where my preschool kids play actively are safe.^a^			0.83					0.85		
The outdoor areas in my neighborhood where my preschool kids can play actively are clean.^e^			0.72					0.80		
**Scale D: Neighborhood Environment Safety**										
I feel safe from crime in my neighborhood and nearby.^a^				0.56					0.68	
I feel safe from biting insects like mosquitos, ticks, and scorpions, and animals like dogs running loose, in my neighborhood and nearby.^a^				0.80					0.82	
**Scale E: Frequency of Active Play Outdoors**										
When the weather is good, how often do your preschool kids usually play actively in outdoor areas like parks, pools, playgrounds, near your home?^d^					0.66					0.78
How often do your preschool kids usually play actively in free or low-cost recreation centers or other indoor places near your home?^d^					0.88					0.83
**Eigenvalue**	2.54	1.62	4.45	1.20	1.29	1.99	2.55	3.94	1.15	1.39
**% Variance**	14.12	8.93	24.72	6.68	7.20	11.06	14.15	21.90	6.36	7.74
**Cronbach’s α**	0.75	0.73	0.89	0.43	0.53	0.68	0.77	0.87	0.52	0.56

^a^Answer choices: Strongly disagree, disagree, neither agree nor disagree, agree, strongly agree; score = 1 to 5, respectively

^b^Number of toys scored 1 = 0 to 4, 2 = 5 to 10, 3 = 11 to 15, 4 = 16 to 20, 5= >20

^c^Number of somersaults scored 1 = <2, 2 = 2, 3 = 3, 4 = 4 to 5, 5= >5 somersaults

^d^Answer choices: Almost never, 1–2 times/week, 3–4 times/week, 5–6 times/week, every day; score = 1 to 5, respectively

^e^Answer choices: Strongly disagree, disagree, neither agree nor disagree, agree, strongly agree, don’t know; score = 1 to 5, respectively and items with “don’t know” answers were omitted from analysis

Five scales were identified from this five factor solution and renamed to more adequately reflect the item content of each scale. The *Indoor Home Space & Supports for Physical Activity* scale has 6-items that assesses the space available inside the home for active play, supports for physical activity inside, and frequency of use. The 4-item *Outdoor*/*Yard Space & Supports for Physical Activity* scale assesses space and supports available immediately outside the home (yard) for active play. The *Neighborhood Space & Supports for Physical Activity* scale has 4-items that assess neighborhood active play space and supports. The 2-item, *Neighborhood Environment Safety* scale assesses perceived environmental safety of active play in the neighborhood. The 2-item *Frequency of Active Play Outdoors* scale assesses how often preschool kids play actively in outdoor areas and in nearby recreation centers or other indoor areas. All items are scored using a 5-point Likert scale (strongly disagree to strongly agree), except frequency of use items which have a 5-point scale ranging from almost never to everyday. The *Neighborhood Space & Supports for Physical Activity* scale also has a “don’t know” answer option in case parents are not aware of neighborhood resources. Scale scores are calculated by averaging responses to all items in the scale, except “don’t know” answers which are not scored or averaged in scale scores. Internal consistency measured by Cronbach’s alpha was good for three scales and, given their brevity, acceptable for the two-item scales.

Spearman rank-order correlations were conducted to compare scale scores with physical activity behavior and cognitions, and screen time to establish convergent validity. There were significant positive correlations among nearly all scale scores and parent and child physical activity levels, value parents placed on physical activity for self and child, importance parents placed on not modeling sedentary behaviors, physical activity outcome expectations, and frequency that parents modeled physical activity (Table 4). These strong, positive correlations among behaviors and cognitions and the five scales indicate strong convergent validity of the HOP-Up. Additionally, there were significant negative correlations of parent and child screen time (minutes/day) with a few of the HOP-Up scales such as *Neighborhood Space & Supports for Physical Activity* and *Outdoor*/*Yard Space & Supports for Physical Activity* indicating convergent validity.

**Table 4:** Spearman rank-order correlations of HOP-Up questionnaire scales and physical activity level and cognitions (*N* = 655)

Scale	Parent Physical Activity Level^a^	Child Physical Activity Level^a^	Value Parent Placed on Physical Activity for Self^b^	Value Parent Placed on Physical Activity for Child^b^	Value Parent Placed on Not Modeling Sedentary Behaviors to Child^b^	Physical Activity Outcome Expectations^b^	Frequency Parent Modeled Physical Activity to Child^c^	Parent Screen- time (min/day)^d^	Child TV/Movie Time Allowed (min/day)^d^	Child Computer Time Allowed (min/day)^d^	Child Active Video Game Allowed (min/day)^d^
Indoor Home Space & Supports for Physical Activity	0.19**	0.29**	0.16**	0.32**	0.09*	0.13**	0.26**	0.09*	−0.01	−0.06	0.01
Outdoor/Yard Space & Supports for Physical Activity	0.11*	0.26**	0.17**	0.35**	0.17**	0.26**	0.12**	0.05	−0.08	−0.08*	−0.09*
Neighborhood Space & Supports for Physical Activity	0.06	0.08*	0.06	0.17**	0.17**	0.15**	0.14**	−0.11**	−0.13*	−0.10	−0.08*
Neighborhood Environment Safety	0.18**	0.05	0.23*	0.17**	0.13**	0.13**	0.14**	−0.07	−0.03	−0.07	−0.02
Frequency of Active Play Outdoors	0.35**	0.23**	0.35**	0.29**	0.11**	0.01	0.32**	−0.02	0.04	0.04	0.08*

*p < 0.05

**p < 0.01

^a^ Physical activity level assessed using 3-items adapted from the International Physical Activity Questionnaire 45 (possible score range 0 to 42)

^b^ Responses were on 5-point Likert scales ranging from strongly disagree to strongly agree. Higher scores indicate greater importance placed on physical activity for self, physical activity for child, and not modeling sedentary behaviors, or more positive outcome expectations associated with engaging in physical activity

^c^ Responses were on a 7-point scale ranging from almost never to everyday. Items are averaged for an overall score; higher scores indicate greater frequency of modeling physical activity for child

^d^ Responses of parent and child screen time are from parent reports in minutes per day

Assessment of readability grade level using the Flesch-Kincaid statistic indicated an 8th grade reading level of this 18-item measure. Thus, the HOP-Up questionnaire is readable and understandable by a large proportion of the population, but caution should be exercised when using with lower literacy populations.

### Phase 10 final expert review

A final expert review of the factor solutions generated in phase 9 confirmed scale content validity. Experts agreed that even though the dog ownership was too low to include this item in the factor analysis, dogs are important active play supports that increase physical activity levels when owners exercise with their dogs [47]. Keeping the original dog item (“How often does your child go on walks with the dog or play with it outside [doing things like throwing balls]”?) or incorporating dog:family co-play into other active play support items should be considered for inclusion in the *Outdoor*/*Yard Space & Supports for Physical Activity* scale.

Experts also recognized that even though the item related to availability of active play video games (games that are played standing up and require lots of moving) was eliminated during factor analysis, the availability of this active play support could be captured in the item related to toys available for active play indoors. Similarly, the wheeled toy availability and swings and slides availability items from the *Physical Activity Environment Immediately Outside the Home* (*Yard*) scale was eliminated in factor analysis, but these concepts could be incorporated in the toys supporting active outdoor play item that was retained.

The neighborhood environment safety items related to traffic and crowding were deleted during factor analysis, which experts found somewhat surprising given that numerous studies have reported that high traffic areas and crowded conditions are negatively correlated with physical activity level [21, 48]. However, recent research has found little evidence supporting the associations of perceived crime-related safety with physical activity in other countries [49]. It may be that concepts of traffic and crowding are captured in frequency of use items in that if these conditions exist, engagement in physical activity outdoors may be curtailed and vice versa.

The frequency of indoor play, active play with siblings or playmates, and use of neighborhood recreation centers items also seemed to account for the deletion of items related to limits put on indoor play (*Accessibility Inside the Home*), availability of siblings and playmates (*Availability of Space & Supports Inside the Home*), and recreation center availability (*Availability of Space & Supports in the Neighborhood*) items, respectively. That is, if limits are set, siblings/playmates are unavailable, and recreation centers are unavailable, the frequency of play would decline and vice versa. The migration of two items related to outdoor areas being safe and outdoor areas being clean from *Neighborhood Access & Safety* in phase 8 to the *Neighborhood Space & Supports for Physical Activity* in phase 9 suggests parents perceive “clean” and “safe” as components of neighborhood space and supports. Similarly, the elimination of the item related to outdoor play near the home (*Frequency of Outside Home Play*) yet retention of the analogous version of this item from the *Frequency of Neighborhood Active Play* scale suggests that the location of outdoor play (yard vs. neighborhood area) is less important than the concept of play taking place outdoors.

It was not surprising that the item asking parents to estimate outdoor play space availability in terms of parking space equivalents performed poorly with low agreement between researcher and participant, and was subsequently eliminated in the factor analysis. Although both parents and researchers indicated outdoor areas for active play were equivalent to about 9–10 parking spaces, the parking spaces estimation item required spatial abilities, such as spatial perception, mental rotation, and spatial visualization, which are known to have significant sex differences that favor men [50]. Given parents in this study were predominantly women, they may have had poor skills for this task. Nonetheless, methods that result in accurate play area estimates without imposing participant burden of taking actual measurements should continue to be explored (e.g., the number of giant steps parents can take along the edges of the play area). It also may be that availability of play space above a certain dimension has a saturation point at which it is “big enough” to accommodate active play correlated with preschool child activity.

## Conclusions

This is the first study, to our knowledge, to develop a brief, easy-to-use, self-report, comprehensive questionnaire to evaluate the physical activity space, supports, and frequency of use in and near homes with preschool children and report the extensive, rigorous testing of its psychometric properties. Overall, the reliability and validity of the HOP-Up questionnaire are good. However, study findings are limited in that phase 8 field test participants were mostly female, white, had completed at least some college and lived in the same geographical location. Even though participants in phase 9 were more diverse, they may not be representative of all parents with preschool-aged children due to self-selection biases and limited geographical representation. Further research is needed to replicate the study findings in a more nationally representative population. Additionally, future studies should assess the construct validity of this measure by examining relationships of the HOP-Up to objective measures of weight status [25], such as body mass index (BMI), waist circumference, and percent body fat of parents and BMI-for-age z-scores of children, and objective physical activity measures such as pedometers or accelerometers.

Despite its limitations, this study is the first to field test and validate a comprehensive measure of the home environment that focuses on the inside, immediate outside (yard), and neighborhood physical activity environment of preschoolers using a socioecological approach. The HOP-Up questionnaire also improves previous checklist-type instruments [16, 20, 25] by using Likert scales, expanding them to include neighborhood physical activity environment, and reducing participant burden with the 18-item questionnaire, which is much shorter than existing instruments. Additionally, the HOP-Up underwent extensive and rigorous testing to establish validity (content, face, criterion, convergent) and reliability (test-retest and internal consistency). The development and evaluation of this measure provides researchers with a new tool that may be useful in benchmarking and tracking change in the physical activity environments of families with preschool-aged children. This brief, easy-to use, valid, reliable questionnaire also may help parents identify simple changes they can make to their home environments that promote more physical activity in their preschool-aged children, thereby helping prevent childhood obesity. Additionally, the HOP-Up may be useful for residential planners and city councils when designing, developing, and modifying neighborhoods to promote increased physical activity. Future research is needed to replicate these study findings and strengthen the use and applicability of this measure in obesity prevention interventions and programs.

## Abbreviations

BMI: Body mass index
HOP-Up: Home opportunities for physical activity check-up questionnaire
ICC: Intra-class correlations
